# Substantially different findings concerning the cardiovascular and cerebrovascular effectiveness of GLP1RAs vs. SGLT2is

**DOI:** 10.3389/fcvm.2022.1034095

**Published:** 2022-10-18

**Authors:** Lixin Du, Yu Zhang, Pan Wang, Zhigang Li, Yunhui Zhao

**Affiliations:** Department of Medical Imaging, Shenzhen Longhua District Central Hospital, Shenzhen, China

**Keywords:** glucagon-like peptide 1 receptor agonists, sodium-glucose cotransporter 2 inhibitors, heart failure, cardiovascular, stroke, death

## Introduction

Baviera et al. conducted a cohort study (1) which mainly focused on the effectiveness of glucagon-like peptide 1 receptor agonists (GLP1RAs) vs. sodium-glucose cotransporter 2 inhibitors (SGLT2is) on cardiovascular and cerebrovascular endpoints in patients with type 2 diabetes (T2D). Accordingly, the authors produced their main findings: compared with SGLT2is, GLP1RAs showed significant reductions in the risks of myocardial infarction (MI) [hazard ratio (HR) and 95% confidence interval (CI): 0.77 (0.66, 0.90)], major adverse cardiovascular events-3 [MACE-3: a composite of MI, stroke, or all-cause mortality (ACM)] [HR (95% CI): 0.91 (0.84, 0.98)], and MACE-4 (a composite of MACE-3 or unstable angina) [HR (95% CI): 0.92 (0.86, 0.99)]. On the contrary, GLP1RAs vs. SGLT2is had the similar risks of hospitalization for heart failure (HHF) [HR (95% CI): 1.03, (0.89, 1.15)] and stroke [HR (95% CI): 0.96 (0.75, 1.21)]. Moreover, the intention-to-treat analysis in Baviera et al.'s study (1) suggested that GLP1RAs were significantly associated with the reduced risk of ACM [HR (95% CI): 0.90 (0.82, 0.99)] compared with SGLT2is. However, these findings are substantially different with previous evidences deriving from randomized controlled trials (RCTs) as well as those deriving from cohort studies.

## Substantial differences in MI, MACE, and ACM

Three conventional meta-analyses (2–4) (“conventional” means “non-network meta-analysis”) based on the cardiovascular outcome trials (CVOTs) of GLP1RAs identified that GLP1RAs vs. placebo reduced MI by about 10% [HR (95% CI): 0.90 (0.83, 0.98)], reduced MACE (a composite of MI, stroke, or cardiovascular mortality) by about 14% [HR (95% CI): 0.86 (0.80, 0.93)], and reduced ACM by about 12% [HR (95% CI): 0.88 (0.82, 0.94)]. Meanwhile, three conventional meta-analyses (4–6) based on the RCTs (mainly including CVOTs) of SGLT2is identified that SGLT2is vs. placebo reduced MI by about 10% [HR (95% CI): 0.90 (0.83, 0.98)], reduced MACE by about 12% [HR (95% CI): 0.88 (0.83, 0.93)], and reduced ACM by about 13% [HR (95% CI): 0.87 (0.81, 0.94)]. These findings from conventional meta-analyses indirectly suggest the similar benefits of GLP1RAs and SGLT2is on the three endpoints of MI, MACE, and ACM. Two network meta-analyses (7, 8) based on the CVOTs of SGLT2is and GLP1RAs directly revealed no difference between these two drug classes in the risks of MI [risk ratio (RR) and 95% CI: 1.04 (0.92, 1.19)], MACE [RR (95% CI): 0.98 (0.91, 1.07)], and ACM [RR (95% CI): 1.00 (0.91, 1.11)]. Similarly, a network meta-analysis (9) based on 764 RCTs directly revealed no difference between them in the risks of MI [odds ratio (OR) and 95% CI: 0.95 (0.84, 1.08)] and ACM [OR (95% CI): 0.95 (0.86, 1.06)]. Furthermore, an updated meta-analysis (10) based on the cohort studies comparing SGLT2is with GLP1RAs also revealed no difference between them in the risks of MI [HR (95% CI): 0.95 (0.88, 1.02)], MACE [HR (95% CI): 1.00 (0.95, 1.04)], and ACM [HR (95% CI): 0.95 (0.90, 1.00)]. Taken together, GLP1RAs and SGLT2is can confer similar reductions in the risks of MI (both: 10% reduction), MACE (GLP1RAs: 14% reduction; SGLT2is: 12% reduction), and ACM (GLP1RAs: 12% reduction; SGLT2is: 13% reduction). However, Baviera et al.'s study (1) showed the obvious superiority of GLP1RAs over SGLT2is in reducing the risks of MI, MACE-3, MACE-4, and ACM.

## Substantial differences in stroke and HHF

Three conventional meta-analyses (2–4) based on the CVOTs of GLP1RAs identified that GLP1RAs vs. placebo reduced stroke by about 17% [HR (95% CI): 0.83 (0.76, 0.92)] and reduced HHF by about 11% [HR (95% CI): 0.89 (0.82, 0.98)]. Meanwhile, three conventional meta-analyses (4–6) based on the RCTs (mainly including CVOTs) of SGLT2is identified that SGLT2is vs. placebo had similar stroke risk [HR (95% CI): 0.99 (0.88, 1.11)] and reduced HHF by about 31% [HR (95% CI): 0.69 (0.65, 0.72)]. These findings from conventional meta-analyses indirectly suggest the superiority of GLP1RAs over SGLT2is in reducing stroke and the superiority of SGLT2is over GLP1RAs in reducing HHF. Two network meta-analyses (7, 8) based on the CVOTs of SGLT2is and GLP1RAs directly revealed the superiority of SGLT2is over GLP1RAs in reducing HHF [RR (95% CI): 0.76 (0.68, 0.85)], and revealed that only GLP1RAs but not SGLT2is reduced stroke. Similarly, a network meta-analysis (9) based on 764 RCTs directly revealed that SGLT2is reduced more HHF events than GLP1RAs [OR (95% CI) of SGLT2is vs. GLP1RAs: 0.74 (0.65, 0.85)], and GLP1RAs reduced more stroke events than SGLT2is [OR (95% CI) of SGLT2is vs. GLP1RAs: 1.20 (1.03, 1.41)]. Furthermore, an updated meta-analysis (10) based on the cohort studies comparing SGLT2is with GLP1RAs also revealed the superiority of GLP1RAs in reducing stroke [HR (95% CI) of SGLT2is vs. GLP1RAs: 1.10 (1.01, 1.19)] and the superiority of SGLT2is in reducing HHF [HR (95% CI) of SGLT2is vs. GLP1RAs: 0.79 (0.71, 0.88)]. Taken together, GLP1RAs vs. SGLT2is can reduce more stroke events (GLP1RAs: 17% reduction; SGLT2is: cannot reduce stroke), and SGLT2is vs. GLP1RAs can reduce more HHF events (SGLT2is: 31% reduction; GLP1RAs: 11% reduction). However, Baviera et al.'s study (1) showed no difference between GLP1RAs and SGLT2is in the risks of stroke and HHF.

## Substantial differences in T2D subgroups

Baviera et al.'s study (1) showed that in T2D patients without previous cardiovascular disease (CVD) GLP1RAs vs. SGLT2is were associated with lower risks of MACE-3 [HR (95% CI): 0.87 (0.80, 0.96)], MACE-4 [HR (95% CI): 0.90 (0.83, 0.98)], and ACM [HR (95% CI): 0.88 (0.79, 0.98)]; and similar risk of HHF [HR (95% CI): 1.03 (0.87, 1.23)]. On the contrary, Lin et al.'s network meta-analysis (8) showed that in T2D patients without previous CVD SGLT2is vs. GLP1RAs had similar risk of MACE [HR (95% CI): 0.93 (0.73, 1.19)]. Moreover, a meta-analysis (11) focusing on the cardiovascular and cerebrovascular effectiveness of SGLT2is vs. GLP1RAs in T2D patients according to CVD status showed that in CVD-free T2D patients SGLT2is vs. GLP1RAs were associated with lower risk of HHF [HR (95% CI): 0.77 (0.67, 0.90)], and similar risk of ACM [HR (95% CI): 0.99 (0.89, 1.10)]. On the other hand, Baviera et al.'s study (1) showed that in T2D patients with previous CVD GLP1RAs vs. SGLT2is had similar risks of MI [HR (95% CI): 0.81 (0.62, 1.05)], ACM [HR (95% CI): 0.94 (0.81, 1.10)], stroke [HR (95% CI): 0.89 (0.60, 1.32)], and HHF [HR (95% CI): 1.00 (0.82, 1.21)]. On the contrary, Ali et al.'s meta-analysis (4) showed that in T2D patients with previous CVD SGLT2is reduced more HHF events than GLP1RAs [HR (95% CI) of SGLT2is vs. placebo: 0.68 (0.63, 0.74); HR (95% CI) of GLP1RAs vs. placebo: 0.91 (0.83, 1.00); *P* for subgroup difference < 0.01], and GLP1RAs reduced more stroke events than SGLT2is [HR (95% CI) of GLP1RAs vs. placebo: 0.84 (0.76, 0.94); HR (95% CI) of SGLT2is vs. placebo: 0.99 (0.88, 1.11); *P* for subgroup difference = 0.04]. Moreover, Du et al.'s meta-analysis (11) based on eleven cohort studies showed that in T2D patients with CVD SGLT2is vs. GLP1RAs were associated with lower risks of HHF [HR (95% CI): 0.82 (0.69, 0.97)], MI [HR (95% CI): 0.86 (0.79, 0.94)], and ACM [HR (95% CI): 0.88 (0.81, 0.96)].

## Discussion

In our opinion, there are three possible reasons for the discrepancies between Baviera et al.'s findings and previous evidences. First, there might be obvious differences between Baviera et al.'s study (1) and previous studies in terms of some important cardiometabolic risk factors, such as HbA1c, body mass index, duration of diabetes, proportion of men, and blood pressure level. These differences in risk factors could contribute to the different findings regarding the cardiovascular and cerebrovascular effectiveness of GLP1RAs vs. SGLT2is in Baviera et al.'s study (1) vs. previous studies. However, in all the studies cited in this paper authors adjusted for as many confounders as possible when they calculated the estimators for the relative cardiovascular and cerebrovascular effectiveness of GLP1RAs vs. SGLT2is. Therefore, the first reason is not very important. Second, as shown in Figures 1, 2 in Baviera et al.'s study (1), the 95% CIs for individual cardiorenal outcomes such as MI, stroke, heart failure, and kidney failure were wider than those for the composite outcomes of MACE-3 and MACE-4. This suggested that the statistical power for assessing these composite outcomes was sufficient, whereas that for assessing those separate outcomes might be not. Therefore, the lack in statistical power for MI, stroke, and heart failure could greatly contribute to the different findings regarding these outcomes in Baviera et al.'s study (1) vs. previous studies. Last (this point is most important in our opinion), all the studies cited in this paper only compared overall GLP1RAs with overall SGLT2is, but failed to compare certain specific GLP1RAs with certain specific SGLT2is. However, significant differences in preventing cardiovascular/cerebrovascular events existed among different GLP1RAs and among different SGLT2is (12). Moreover, the efficacy of different GLP1RAs and SGLT2is in preventing MACE varied in different T2D subgroups, such as T2D with or without cardiorenal disease (13). Therefore, the reason why Baviera et al.'s study (1) identified substantially different findings concerning the cardiovascular/cerebrovascular effectiveness of GLP1RAs vs. SGLT2is compared with previous studies is probably that the components of the intervention measure (i.e., GLP1RAs) and/or the control measure (i.e., SGLT2is) were different among these studies. However, we failed to confirm it due to the limited data provided in these studies. Therefore, it is meaningful for future studies to address this issue.

In summary, both previous evidences deriving from RCTs (especially, CVOTs) and those deriving from cohort studies suggest the similar benefits of GLP1RAs and SGLT2is on the three endpoints of MI, MACE, and ACM; but the superiority of GLP1RAs over SGLT2is in reducing stroke, and the superiority of SGLT2is over GLP1RAs in reducing HHF. Oppositely, Baviera et al.'s study (1) shows that GLP1RAs vs. SGLT2is were associated with lower risks of MI, MACE-3, MACE-4, and ACM; but similar risks of stroke and HHF. Moreover, there are also significant differences between previous evidences and Baviera et al.'s findings in terms of the cardiovascular and cerebrovascular effectiveness of SGLT2is vs. GLP1RAs in T2D patients according to CVD status. The possible reasons for these substantial discrepancies were discussed in detail in this paper and would prompt more meaningful studies.

## Author contributions

LD wrote the manuscript. YZhan, PW, ZL, and YZhao reviewed the manuscript. All authors approved the manuscript.

## Funding

This work was supported by the Key Laboratory of Neuroimaging, Longhua District, Shenzhen [Shen Long Hua Ke Chuang Ke Ji Zi (2022) No. 7]; and Shenzhen Fundamental Research Program (Natural Science Foundations), General Program for Fundamental Research (Grant No. JCYJ20210324142404012).

## Conflict of interest

The authors declare that the research was conducted in the absence of any commercial or financial relationships that could be construed as a potential conflict of interest.

## Publisher's note

All claims expressed in this article are solely those of the authors and do not necessarily represent those of their affiliated organizations, or those of the publisher, the editors and the reviewers. Any product that may be evaluated in this article, or claim that may be made by its manufacturer, is not guaranteed or endorsed by the publisher.
